# The Role of Portable Incisional Negative Pressure Wound Therapy (piNPWT) in Reducing Local Complications of Post-bariatric Brachioplasty: A Case-Control Study

**DOI:** 10.1007/s00266-020-02122-1

**Published:** 2021-01-22

**Authors:** Federico Facchin, Andrea Pagani, Paolo Marchica, Laura Pandis, Carlotta Scarpa, Tito Brambullo, Franco Bassetto, Vincenzo Vindigni

**Affiliations:** 1 Plastic and Reconstructive Surgery Unit, University of Padova, Via Nicolò Giustininani 2, 35128 Padua, Italy; 2 Clinic and Policlinic of Plastic and Hand Surgery, Technical University of Munich, Ismaninger Str. 22, 81675 Munich, Germany

**Keywords:** Body-contouring, Post-bariatric patients, Surgical site complications, Obesity, Brachioplasty, Wound care

## Abstract

**Background:**

Due to the great impact of bariatric surgery on the overweight epidemic, the number of post-bariatric body-contouring procedures is constantly increasing worldwide. The portable incisional negative pressure wound therapy (piNPWT) is a promising medical device for accelerating wounds closure and controlling post-operative complication, which have been shown promising results in post-bariatric population. We aimed to evaluate the role of piNPWT in optimizing wound healing and controlling post-operative complications after a post-bariatric brachioplasty.

**Patients and Methods:**

26 post-bariatric female patients who underwent a brachioplasty followed by either a piNPWT (14 cases) or a standard wound treatment (12 controls) were analyzed. The number of post-operative dressing changes, the rate of local post-operative complications (re-operation, hematoma and serosa development, dehiscence and necrosis), the time to dry as well as the scar quality and hospitalization length were evaluated.

**Results:**

None of the patients prematurely stopped treatment with piNPWT due to intolerance. The piNPWT patient group showed a significant lower healing time as well as a significant reduction of the number of post-operative dressing changes and hospital stay. Despite the scarring process was excellent from the functional point of view in the long term, we noticed a higher rate of hyperchromic scarring at 90 days after surgery.

**Conclusion:**

The piNPWT is a cost-effective and user-friendly medical tool that increase and promote wound healing. We suggest the use of this device in post-bariatric patients who undergo a brachioplasty, especially if there is the need to minimize the number of post-operative dressing changes.

**Level of Evidence IV:**

This journal requires that authors assign a level of evidence to each article. For a full description of these evidence-based Medicine ratings, please refer to Table of Contents or the online Instructions to Authors www.springer.com/00266.

## Background

The epidemic of overweight and obesity represents a significant threat for patients’ health and a major challenge for the healthcare system [1]. Bariatric surgery and diet-related weight loss reduce significantly comorbidity of obese patients improving their life expectancy [2, 3]. However, the accumulation of excess cutaneous tissue severely impacts massive weight loss (MWL) patients’ quality of life, preventing them from performing social and physical activity. In addition, patients frequently develop skin fold cutaneous infection [4, 5]. More of 30% of the American population is obese and almost 300,000 patients per year undergo bariatric surgery [6, 7].

Drastical changes in the BMI modify the structure of soft tissues, leading progressively to excess skin in different body districts [4, 8–10]. The combination of adiposity and/or poor skin tone with increasing skin ptosis guides surgeons in choosing the best technique according to the Pittsburgh Rating Scale. Patients with loose and hanging skin should undergo a brachioplasty procedure associated with liposuction in the presence of severe adiposity [8, 11, 12]. Some authors suggest that a chest extension of skin resection is often required to obtain a smooth axillary profile [13]. In 2017, 18033 arm lift procedures have been performed in the U.S., with a 5235% increase from 2000 [14].

Among different body areas, the postero-medial region of the arms represents a particular region where adipo-cutaneous excess of MWL patient accumulate in the distal half due to the progressive loosening of the support of superficial, longitudinal and axillary fascial systems of the proximal half [15].

In addition, surgical scars of brachioplasty are the most commonly exposed by every day clothing if compared to other procedures, representing the heavier surgical burden.

Despite surgery is constantly improving, minor and major complications including seroma, hematoma, infection, lymphocele, numbness, peripheral nerve pain and wound dehiscence are still frequent. Post-operative complications rates are as high as 40% in many studies, especially in associated multiple procedures (e.g., abdominoplasty, mastopexy) [6, 7].

Given the steep learning curve and the high risks, body-contouring procedures should be performed in dedicated centers with managed through defined protocols from experienced surgeons.

From the surgical point of view, the deep investing fascia, which envelopes arm musculature, should not be violated during surgery, in order to avoid medial antebrachial cutaneous nerve injuries. An axillary Z-plasty or its sinusoidal variation, can be performed to the proposed skin excision in order to prevent severe contractures and to restore the axillary dome’s appearance. Tension free closure should be obtained. Moreover, intraoperative infiltration of diluted epinephrine and liquid warming have been shown to reduce seroma rate formation and intraoperative bleeding [16].

The preoperative management is of paramount importance in preventing complications with metabolic and nutritional homeostasis achieved before surgery. A stable weight and nutritional evaluation should be granted [12]. Finally, a dedicated post-operative management of wide surgical wounds is of key importance to improve the final result.

At present, the piNPWT has been shown effective in improving wound healing in several surgical fields, reducing at the same time risks of complications even in other surgeries: inguinal dissection [17], mastectomy [18], ALT flap donor site [19], cesarean sections in high-risk patients [20, 21]. Benefits from the piNPWT application have been reported in a case-control study in post-bariatric abdominoplasty [21] and in cost-utility analysis [22].

Altogether, by assuming that this dressing could act as effective, user-friendly and cost-effective, we compared the effect of piNPWT with a traditional dressing change in MWL brachioplasty patients.

## Patients and Methods

The study was conducted in accordance with the World Medical Association Declaration of Helsinki (June 1964) and subsequent amendments.

**Fig. 1:**
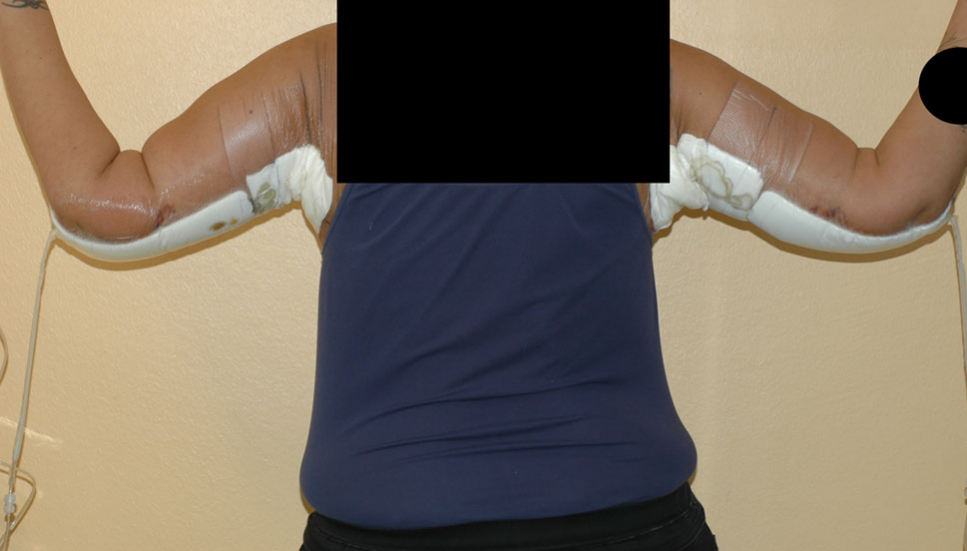
Post-operative dressing with bilateral piNPWT (piNPWT patients)

We performed a case-control study with 26 post-bariatric female patients (middle age 45.33) who underwent a brachioplasty at the Division of Plastic Surgery, at our University-Hospital, from June 2018 to March 2020. Fourteen patients (middle age 49.33) were treated post operatively with bilateral piNPWT (piNPWT patients) Fig. 1 (PICO, Smith and Nephew, Watford, UK) with a negative aspiration pressure of −80mmHg. Twelve patients (middle age 41.66) were treated with a traditional dry dressing (TDD group).

Inclusion criteria:

- Massive weight loss at least of 7 points BMI (post MWL surgery or diet reduction)
- Stable weight and metabolic/nutritional homeostasis for at least 6 months [12]
- Female

Exclusion criteria:

- Unstable weight loss
- Previous arm surgery
- Allergy to glue and tape
- Neurologic, psychiatric or vascular disorders of the upper extremity
- Lymphedema of the arms
- Unrealistic patient expectations
- Raynaud’s disease, connective tissue disorders and advanced rheumatoid arthritis [13, 23–25]

Before surgery, a physical examination was performed, evaluating upper limb function, including ROM at shoulder/elbow/hand and grip strength. Authors assessed for excess fat and skin, overall skin quality and tone.

A watertight layered and subcuticular tension free suture was obtained, and a drain per arm was placed in all patients. Both groups used compressive sleeve garments for 30 days after treatment [16]. Dressings were evaluated daily during the hospital stay. Whereas piNPWT was changed after 7 days post operatively, traditional dressings were changed every 3 days after treatment according to our protocol. Patients were instructed to return for follow-up at 90 days.

During every single dressing, the presence of post-operative complications (i.e., blistering, hematoma, serosa, hypertrophic or hyperchromic scars), the number of post-operative dressing change, the hospitalization length and the eventual impact of the associated surgeries were evaluated. Complications were calculated per arm, defined as either hematoma, wound dehiscence, skin necrosis or infection, and classified through Clavien-Dindo classification [26].

**Fig. 2:**
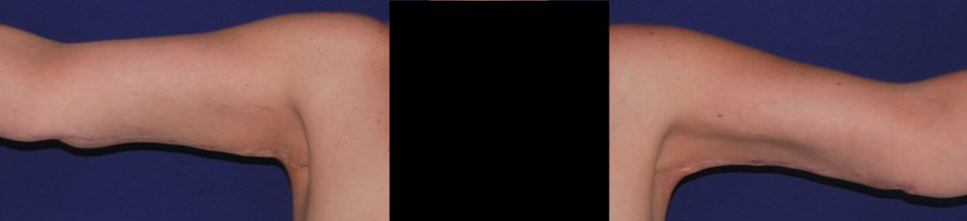
Post-operative long-term (10 months) follow up of PTT group patient

**Fig. 3:**
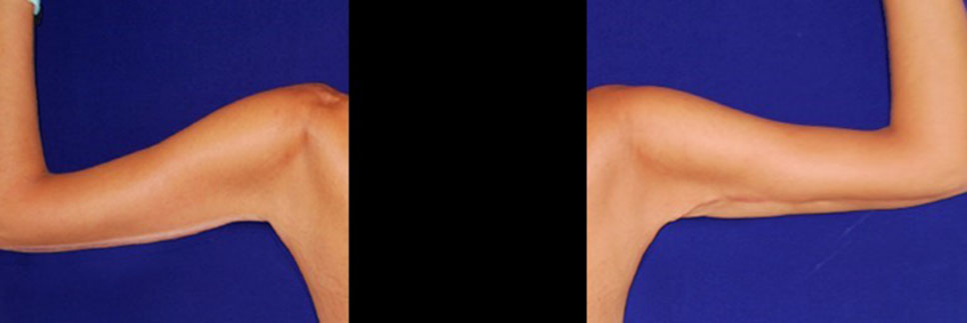
Post-operative long-term (11 months) follow up of piNPWT group patient

The aspect of the scar aspect was evaluated at a mean time of 6 months after surgery with Vancouver Scar Scale [27] (Figs 2, 3).

All procedures have been done in agreement with the declaration of Helsinki.

### Statistical Analysis

Sample size was calculated using Pearson’s chi-square test to detect meaningful differences (alpha 0.05; 1-β: 90%; enrollment ratio (1) between the two groups. Anticipated effect for the outcome (−50%) was based on previous experience and literature on the use of piNPWT in abdominoplasty. Results are expressed as mean ± SD. An unpaired Student’s t Test (GraphPad Prism 7.0, Inc., La Jolla, CA, USA) was used to determine significant differences between groups (*p* value < 0.05).

## Results

**Fig. 4:**
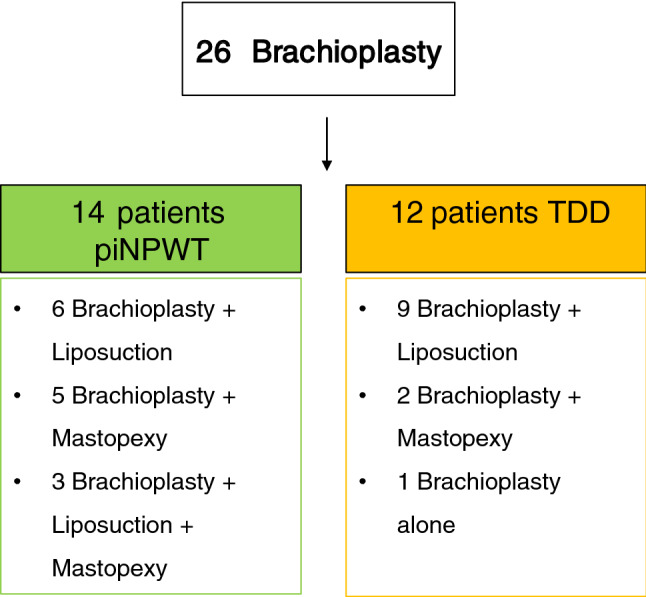
Study design and groups composition

**Fig. 5:**
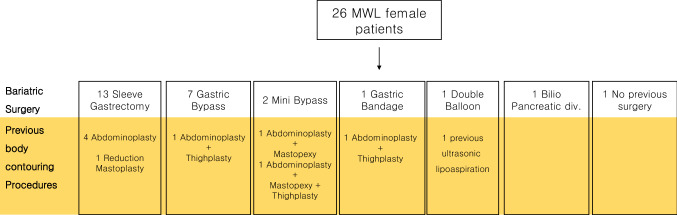
Bariatric surgery and previous body-contouring procedures performed

**Fig. 6:**
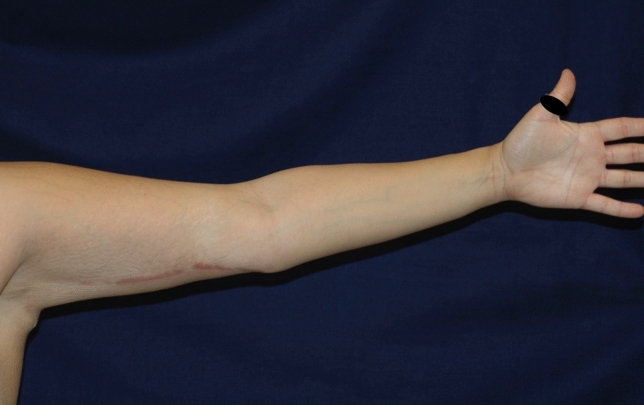
Post-operative result 90 days after surgery in a patient of the piNPWT group who developed an hyperchromic scar

After successfully performing 26 brachioplasty, *n*=14 patients were randomly treated post operatively with a piNPWT and *n*=12 patients with a traditional dressing. (Fig. 4). Among the investigated patients, we did not observe significant differences in demographics as reported in Table 1 except for BMI loss, which appeared to be higher in the piNPWT group. Data regarding bariatric procedures and previous post-bariatric procedures are reported in Fig. 5. 38,4% of patients had a previous body-contouring procedure. Two patients have lost weight without surgery, one of them was excluded from the study because affected by anorexia. Post-operative results and analyzed outcomes are collected in Table 2. As reported in Figs 4, 6, patients of the piNPWT group were treated with brachioplasty and liposuction, 8 patients with the combination of brachioplasty and mastopexy with implant (Polytech Health & Aesthetics Altheimer Str. 32, 64807 Dieburg, Germany) with three of them managed with arm liposuction as well. Figure 4. No intra-operative complications were reported; the piNPWT was generally well tolerated.

**Table 1:** Patients demographics of the two groups

	piNPWT	TDD
Sex	All females	
Age (years) *	43 ± 11.09	49 ± 9.32
Smokers	3	4
Number of obese patient per group BMI > 30	3	5
Time from bariatric procedure (years) *	3.25 ± 2.51	5 ± 4.22
Weight loss (kg)*	54.85 ± 19.73	43.47 ± 17.80
BMI before bariatric surg*	47.52 ± 7.82	45.04 ± 4.55
BMI after bariatric surgery*	27.03 ± 2.91	28.92 ± 4.6
BMI loss*	20.49 ± 6.27	16.12 ± 6.00
Ptosis Degree Pittsburg Rating Scale [8] (El Khatib [13])	5 patients grade 2 (2b) 9 grade 3 (3)	5 patients grade 2(2b) 7 grade 3 (3)
Weight of tissue removed per arm (gr)*	180.71±60.94	185.75±75.98

The two groups were comparable when considering the majority of variable analyzed

*p>0.05

**Table 2:** Post-operative outcomes of the two groups

	piNPWT	TDD
Number of post-operative complication	2	6
Claviend-Dindo Grade I	1	3
Claviend-Dindo Grade II	1s	2
Claviend-Dindo Grade III	0	1
Number of SSI	None	None
Hypercromic scars 90 days after surgery	7	0
Post op. dressing change**	2 ± 0.77	4.91 ± 0.79
Time to dry (days)**	9.36 ± 2.15	17.66 ± 4.79
Hosp. Length (days)**	3.07 ± 114	5.33 ± 1.49

**p < 0.05%

As for post-operative complications, one patient of the TDD group developed a wound dehiscence of the right arm suture requiring surgical revision (Clavien-Dindo Grade III) and concomitant blistering of the left arm treated with wet to dry dressing (Clavien-Dindo Grade II). One patient of the piNPWT group developed blistering treated with wet to dry dressing (Clavien-Dindo Grade II). Two patients of the TDD group developed ecchymosis (Clavien-Dindo Grade I). Two hematomas were treated conservatively in the piNPWT and one in the TDD group (Clavien-Dindo Grade I). The patient with hematoma in the TDD group subsequently developed a seroma requiring multiple aspirations (Clavien-Dindo Grade II).

No reported discomfort was associated with the use of piNPWT or dry dressing. Three patients of the piNPWT group reported increased itching that did not require dressing change or suspension of therapy.

The number of post-operative dressing changes was 4.91 ± 0.79 in the TDD group vs. 2 ± 0.77 in the piNPWT group (*t*-Test=4.24×10^−9^; *p*<0.05). Furthermore, a significant difference was calculated in the “Time to dry” 17.66 ± 4.79 days in the TDD group vs. 9.36 ± 2.15 days in the piNPWT group (*t*-Test=1.52×10^−5^; *p*<0.05). Finally, a significant difference in the hospitalization length was recorded 5.33 ± 1.49 days in the TDD group vs. 3.07 ± 1.14 days in the piNPWT group (*t*-Test=0.0002; *p*<0.05). Seven patients of the piNPWT group developed a hyperchromic scar at 90 days Fig. 6, which then subsides with proper scar management (sun protection and scar massage). The scar clearing was appreciated in subsequent follow-up visits at six, twelve months.

The aspect of the scar was comparable in the two groups with a mean value of the Vancouver Rating Scale at six months of 4.17 ± 1.99 and 4.07 ± 2.49 for the TDD and piNPWT groups, respectively (*p* value > 0.05) (Figs 2, 3).

## Discussion

MWL patients are at higher risk for developing complications if compared to standard population. Obesity, smoking history and anemia are known risk factors that commonly impair body-contouring procedures outcomes [28]. In addition, according to our previous experience, BMI greater than 30 kg/m^2 ^appeared to be negative influence scarring process. Furthermore, concomitant body-contouring procedures seem to increase post-operative complications [29]. Greater BMI loss has been reported as risk factor for the need of longer incisions and operative time [30].

The present study focused on brachioplasty as one of the main surgeries requested by post-bariatric female patients and more frequently burdened by complications related to wound healing and scar quality.

Our outcomes are consistent with those reported for other field of surgery and post-bariatric abdominoplasty as well, confirming the role of piNPWT to manage high risk surgical wounds [19, 21, 22, 31]. This study suggests that a user-friendly device applied under garments adequately limits the number of complications without eliciting treatment-related cutaneous damage or causing patient discomfort responsible of premature dressing change. However, no beneficial effect was observed in preventing major complication Clavien-Dindo grade III [26]

The ability of the piNPWT to reduce the lateral tense of closed incision improved wound healing in our patients [32]. The role of the piNPWT in enhancing the reabsorption of tumescent solution after liposuction should be further investigated in MWL patients treated with the combination of lifting and liposuction.

The combination of body-contouring procedures is considered a risk factor for potential increasing of complication. Even if the combination of mastopexy and brachioplasty is a general favorable combination of procedures, the decision should be made evaluating patient’s expectations, BMI loss, comorbidities and smoking status [33].

Among our groups, the higher number of patients undergoing a combination of mastopexy and brachioplasty in piNPWT sample (8 vs. 2) may have negatively influenced the post-operative course of the patients and the wound healing process. On the contrary, the portable incision NPWT may have lessen the risk. Although the scarring was from the functional point of view extremely satisfactory and comparable in both groups, according to our previous experience [29], the hyperchromic scar is an aesthetic aspect that should not be underestimated, especially in post-bariatric female patients.

VL Young et al. [34] indicates that patients are highly concerned about scarring following routine surgery. Their work also shows that there are disparities in patient-clinician communication regarding expectations following surgery. Hence, patients should be accurately counsel, given the transient effect on hyperchromic scars, which can negatively influence from a psychological point of view the post-operative period of MWL patients [26].

The reduced number of post-operative dressing changes and shorter extent of the hospital stay of piNPWT patients together with the reduction of local complications is known to impact favorably the cost management of this group of patients [22, 35].

Nonetheless, the presence of two dressing devices constantly attached to patients’ arms could be annoying for patients. Even though, accordingly to the literature, nobody of piNPWT group complained about their dressing, patients should be accurately informed about the need to carry two devices hung to their arms for 7–12 days[36].

In addition, in the middle of the COVID-19 era, the reduction of the hospital stay, number of outpatient visits and complications allow limiting the exposure of patients to the risk of Sars-Cov2 infection [37, 38].

Our preliminary report, even if retrospectively, confirms the role of piNPWT in improving postoperative management of post-bariatric patient undergoing body-contouring procedures. Other comparative studies are needed to validate this approach in breast lifts and thigh lift. Further research has to be carried out before routine clinical adoption of this technique in particular to find the ideal group of patients that will benefit most (i.e., combination of body-contouring procedures or patient undergoing liposuction and lifting).

As people heal differently, a prospective comparison of the effect of the application piNPWT in one arm and traditional dressing in the other arm of a single patient could be an option to limit inter-patients variability.

Furthermore, a more comprehensive analysis of the cost-effectiveness of the procedure should also be conducted.

## Conclusions

In conclusion, we demonstrate that piNPWT is an effective medical tool, able to limit minor local complications rates in post-bariatric patients undergoing a brachioplasty.

The piNPWT represents a valid alternative to the traditional dry dressing. Given the transiently worrisome aesthetic result, informing the patient about the possible development of a hyperchromic scar is key. We suggest the use of this device in post-bariatric patients who undergo a brachioplasty, especially if there is the need to minimize the number of post-operative dressing changes or outpatients’ visits and when brachioplasty is combined with liposuction and/or mastopexy.
